# APOBEC3G Inhibits Elongation of HIV-1 Reverse Transcripts

**DOI:** 10.1371/journal.ppat.1000231

**Published:** 2008-12-05

**Authors:** Kate N. Bishop, Mohit Verma, Eun-Young Kim, Steven M. Wolinsky, Michael H. Malim

**Affiliations:** 1 Department of Infectious Diseases, King's College London School of Medicine, Guy's Hospital, London, United Kingdom; 2 Division of Infectious Diseases, Northwestern University Feinberg School of Medicine, Chicago, Illinois, United States of America; Northwestern University, United States of America

## Abstract

APOBEC3G (A3G) is a host cytidine deaminase that, in the absence of Vif, restricts HIV-1 replication and reduces the amount of viral DNA that accumulates in cells. Initial studies determined that A3G induces extensive mutation of nascent HIV-1 cDNA during reverse transcription. It has been proposed that this triggers the degradation of the viral DNA, but there is now mounting evidence that this mechanism may not be correct. Here, we use a natural endogenous reverse transcriptase assay to show that, in cell-free virus particles, A3G is able to inhibit HIV-1 cDNA accumulation not only in the absence of hypermutation but also without the apparent need for any target cell factors. We find that although reverse transcription initiates in the presence of A3G, elongation of the cDNA product is impeded. These data support the model that A3G reduces HIV-1 cDNA levels by inhibiting synthesis rather than by inducing degradation.

## Author Summary

APOBEC proteins are cell-encoded factors that inhibit the replication of numerous retroviruses, such as HIV-1, and retrotransposons. In many cases, inhibition is clearly associated with cytidine-to-uridine editing of viral or transposon DNA. On the other hand, a number of studies with particular APOBEC protein/substrate combinations, or engineered proteins that are editing-deficient, have indicated that inhibitory mechanism(s) distinct from editing are also operative. Here, we have analyzed the effects of APOBEC3G, a potent HIV-1 inhibitor, on viral reverse transcription using cell-free viruses (natural endogenous reverse transcriptase assays). We report that APOBEC3G inhibits viral DNA synthesis in a dose-dependent fashion, and does not require editing capabilities to do so. Because the addition of the first nucleotide to the tRNA primer is unaffected by A3G and the magnitude of inhibition increases as later reverse transcription intermediates are measured, we suggest that APOBEC3G acts by impeding the translocation of the reverse transcriptase enzyme along its RNA template, perhaps by binding directly to the RNA. These results provide novel insight into the biological activities of this class of host anti-viral proteins.

## Introduction

APOBEC3G (A3G) is a potent anti-viral polynucleotide cytidine deaminase, initially identified as the cellular target of the HIV-1 viral infectivity factor (Vif) protein [1]. Since this discovery, many APOBEC proteins from various species have been shown to inhibit the replication of a diverse range of viruses and retrotransposons (see [2]–[4] for reviews). However, the exact mechanism(s) by which APOBEC proteins elicit these effects is unresolved, enduringly controversial, and may differ among APOBEC family members.

Studies on HIV-1 have revealed that in the absence of the Vif protein, human A3G from virus-producing cells is packaged into HIV-1 particles [5]–[9]. Two phenotypes are then observed when these particles infect new target cells: first, nascent viral reverse transcripts are extensively mutated [10]–[12]. Most of the mutations detected are G-to-A changes in the positive sense, coding strand, implying that deamination of cytidines must occur predominantly on the negative sense strand of cDNA. This, in turn, locates A3G to the site and time of reverse transcription. The fixation of such hypermutation in proviral sequences would presumably lead to the expression of inactive or truncated viral proteins, producing non-infectious virions. The second observed phenotype is a reduction in the accumulation of viral cDNA in target cells [11], [13]–[20]. This could occur either by triggering degradation of reverse transcripts or by inhibiting DNA synthesis.

We, and others, initially proposed a mechanism that linked hypermutation to DNA degradation [10],[11]. Specifically, the hypothesis was that cellular enzymes known as uracil DNA glycosylases would recognise and remove uracil in the viral cDNA leaving abasic sites. These sites would then be cleaved by cellular apurinic/apyrimidinic endonucleases resulting in the degradation of single-stranded reverse transcripts. However, recent studies have challenged this theory. Several groups have now shown that knocking out or inhibiting the cellular uracil DNA glycosylases UNG2 and SMUG1 does not rescue the defect in viral cDNA accumulation [16],[17],[21]. Furthermore, there is mounting evidence to suggest that hypermutation can be dispensable for APOBEC-mediated antiviral activity (reviewed in [3]). For example, various APOBEC proteins are able to inhibit MMTV, HBV, AAV or retrotransposons with little or no discernible editing activity [22]–[24]; A3G expressed in unstimulated CD4+ T-cells can inhibit incoming HIV-1 without inducing widespread hypermutation [25]; levels of mutations detected in HIV-1 do not correlate with the degree of viral inhibition or cDNA levels [13],[21]; and finally, engineered catalytically inactive APOBEC mutants can still inhibit HIV-1, MMTV, HBV or retrotransposons in cultured cell experiments [14],[23],[24],[26],[27]. In the absence of editing, therefore, what would be the trigger for viral cDNA degradation?

Alternatively, it is plausible that APOBEC proteins, which are known to bind both RNA and single stranded DNA [28]–[30], may be able to prevent the synthesis of cDNA by interfering with the process of reverse transcription. In this regard, various conflicting reports have suggested that A3G is able to inhibit numerous steps during HIV-1 replication, including primer tRNA annealing, minus and plus strand transfer, primer tRNA processing and removal, DNA elongation, and proviral integration [11], [13]–[20],[31],[32]. It is possible that minor blocks at any (or all) of these steps could, together, accumulate to produce the potent overall inhibition of HIV-1 infectivity. In addition, it has recently been reported that the observed block to HBV replication is not due to A3G-mediated DNA degradation, but instead due to an inhibition of HBV early minus strand DNA synthesis [33],[34].

We have previously shown that A3G and A3F can elicit a pronounced block to early viral DNA accumulation and that this decrease correlates well with the block to viral infectivity although these proteins induce very different levels of hypermutation during HIV-1 infection [13],[14],[35]. We therefore decided to employ an alternative methodology to examine the effects of APOBEC proteins on the early steps of reverse transcription more closely, namely using using viral particles isolated from cell supernatants to study natural endogenous reverse transcription (nERT). We show here that A3G-mediated inhibition of cDNA accumulation is independent of both the target cell and hypermutation. Using this assay system, we also show there is no defect in tRNA^lys3^ priming of reverse transcription in virions. However, a small decrease in the amount of cDNA can already be observed 16 bases after the start of reverse transcription, and this reduction amplifies with distance from the tRNA^lys3^ primer. The block to cDNA titrates with A3G concentration and can be induced by endogenous levels of A3G. Together with existing data, our results support the proposal that A3G inhibits the elongation of HIV-1 DNA by reverse transcriptase, probably by steric hindrance, rather than promoting the degradation of viral cDNA.

## Results

### Inhibition of cDNA Accumulation Is Independent of Target Cell Type

**Figure 1:**
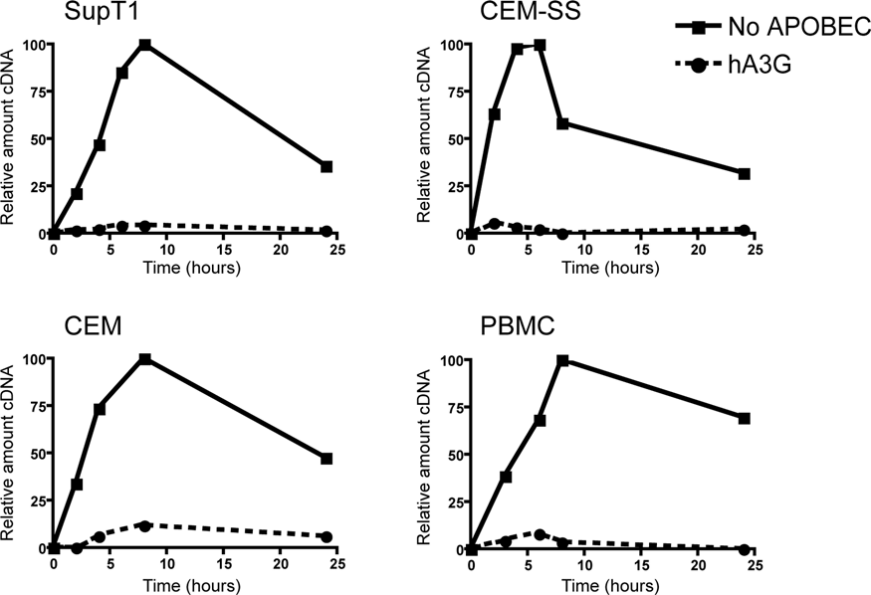
A3G inhibits HIV-1 cDNA accumulation regardless of target cell type. Equivalent amounts of HIV-1/Δ*vif* produced from 293T cells in the presence or absence of human A3G were used to infect SupT1, CEM-SS, CEM, or PBMC target cells. Total DNA was harvested at the indicated times after infection, and the relative amounts of early reverse transcription products (strong stop) were measured using quantitative PCR analysis. Levels of cDNA are shown as percentages of the peak accumulation detected in the control (no APOBEC) reaction. Representative data from at least two independent experiments with each cell type are shown.

We have previously shown that human A3G and A3F inhibit the accumulation of early (strong stop) cDNA products during HIV-1 infection of SupT1 cells (a T-cell line) [13],[14]. It has been proposed that this is due to specific degradation of viral cDNA by target cell enzymes [10],[11]. We therefore wanted to test the effects of A3G on cDNA accumulation in different target cells. We synthesized *vif*-deficient (Δ*vif*) HIV-1 in the presence or absence of A3G in 293T cells and used this virus to infect three different human T-cell lines or peripheral blood mononuclear cells (PBMCs). In case the expression of endogenous A3G affected the accumulation of viral cDNA, we chose two so-called permissive T-cell lines that express little or no endogenous A3G, SupT1 and CEM-SS, and one cell line, CEM, known to express A3G and be non-permissive for HIV-1/Δ*vif* replication [1],[35],[36]. PBMCs include CD4+ T-cells, the natural targets of HIV-1 infection, which also express A3G. Cells were harvested at different times after infection and the relative amounts of HIV-1 strong stop cDNA were measured by quantitative (q)PCR. As seen previously, in the absence of A3G, the level of reverse transcription products increased with time to a peak around 8 h and then declined (Figure 1, filled lines). The levels of transcripts detected in cells infected with virus produced in the presence of A3G were very low at all time points and only just detectable, regardless of which target cell culture had been infected (Figure 1, dotted lines). At the peak of accumulation, the amount of cDNA from these viruses ranged from ∼2–10% of the level of cDNA from control viruses made in the absence of A3G. There was no significant difference between PBMCs and T-cell lines, or between permissive and non-permissive cell types, indicating the inhibition of cDNA accumulation under these experimental conditions is independent of endogenous A3G in the target cell, and can occur in a range of different T-cells.

### Target Cell Proteins Are Dispensable for the Block to cDNA Accumulation

**Figure 2:**
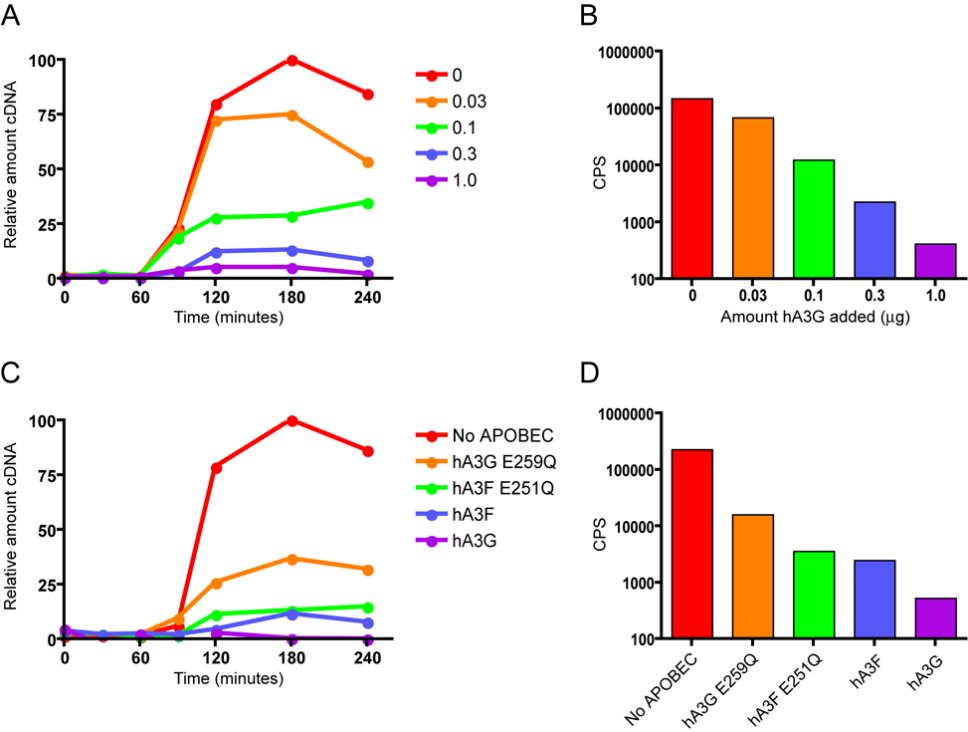
A3G inhibits HIV-1 cDNA accumulation in the absence of target cells and hypermutation. (A) HIV-1/Δ*vif* viruses were generated from 293T transfected with a fixed amount of proviral plasmid (3 µg) and increasing amounts of A3G plasmid (from 0 to 1 µg). Viruses were subjected to natural endogenous reverse transcription reactions, and DNA was harvested at the indicated times after the addition of dNTPs. The relative amounts of early reverse transcription products (strong stop) were measured using quantitative PCR analysis and are represented as in Figure 1. (B) The virus preparations described in (A) were used in parallel to challenge TZM β-gal indicator cells, and productive infection was measured after 24 h as the induction of β-galactosidase activity, monitored using a chemiluminescent substrate. Infectivity is reported as counts per sec. (C) HIV-1/Δ*vif* viruses were produced from 293T cells in the presence of either control vector, wild-type (WT) A3G, WT A3F, or A3G/A3F variants with the catalytic glutamic acid residue altered to a glutamine. Viruses were subjected to natural endogenous reverse transcription reactions, and DNA was harvested at the indicated times after the addition of dNTPs. The relative amounts of early reverse transcription products (strong stop) were measured using quantitative PCR analysis. (D) The virus preparations described in (C) were used in parallel to challenge TZM β-gal indicator cells, and viral infectivity was measured as in (B). All experiments were performed with at least two independent sets of viruses, representative results are shown.

As the A3G-mediated inhibition of viral cDNA accumulation seemed to be independent of the type of target cell infected, we wondered whether any target cell factors were required for this effect at all. Previous studies have tested the contribution of particular cellular proteins to the antiviral activity of A3G by knocking down individual proteins, specifically uracil DNA glycosylases [16],[17],[21]. By employing a nERT assay where cell-free viral particles are incubated in the presence of dNTPs and a membrane pore-forming substance called melittin *in vitro* [37],[38], we could test the involvement of all target cell proteins on A3G's influence on viral cDNA accumulation at once. Under these conditions, reverse transcription of the viral RNA proceeds using the natural tRNA^lys3^ primer and virion reverse transcriptase enzyme. We carried out nERT reactions on HIV-1/Δ*vif* produced in 293T cells in the presence of increasing concentrations of A3G, and measured the amount of strong stop cDNA present at various times after the start of the reaction by qPCR (Figure 2A). As seen with infections of target cells, the amount of viral cDNA increased with time in all reactions, peaking around 3 h after the addition of nucleotides. Strikingly, the amount of strong stop DNA that accumulated decreased with increasing A3G concentration. Virions made in the presence of the highest dose of A3G (1 µg transfected plasmid; Figure 2A, purple line) only accumulated ∼5% of the amount detected in virions made in the absence of A3G (Figure 2A, red line). This was very similar to the decreases in strong stop cDNA seen in T-cell cultures when viruses were made in the presence of 1 µg of A3G plasmid (Figure 1), suggesting that additional factors present in target cells are not required for this defect. The decrease in cDNA accumulation also notably correlated with decreasing viral infectivity as measured in a single cycle infection of TZM β-gal reporter cells (Figure 2B). Similar results were also obtained using an HIV-1 vector system (data not shown), confirming that this phenotype is not specific for the strain of HIV-1 used. Accordingly, because target cell proteins are dispensable for the A3G-mediated suppression of reverse transcript accumulation, we considered the nERT assay to be a simpler, more defined system with which to study this aspect of APOBEC protein function.

### Hypermutation Is Dispensable for the Block to cDNA Accumulation

To confirm that catalytically inactive APOBEC mutant proteins could also function independently of any target cell proteins, we synthesized HIV-1/Δ*vif* in 293T cells in the presence of either control vector, wild-type (WT) A3G, WT A3F, or A3G/A3F variants with the catalytic glutamic acid residue substituted with a glutamine [14]. These viruses were then subjected to nERT followed by qPCR to detect strong stop cDNA. As above, reverse transcripts accumulated with time to a peak at 3 h (Figure 2C). Again, the levels of cDNA were reduced in viruses made in the presence of APOBEC proteins. Mirroring our previous results in cells [14], the catalytic A3G mutant E259Q inhibited cDNA accumulation to a lesser extent, than WT A3G; to ∼40% of control compared to <1% (Figure 2C, compare purple line with orange line). However, the catalytic A3F mutant E251Q had a very similar effect on cDNA accumulation to WT A3F (Figure 2C, compare green line with blue line). As seen with the A3G titration (Figure 2B), the degree of inhibition of cDNA accumulation closely reflected the inhibition of viral infectivity in TZM cells (Figure 2D). Considering panels A and B against panels C and D, it is remarkable that conditions that impart very similar comparative decreases in cDNA levels also reduce infectivity to similar extents: this point is illustrated by comparing the effects of 0.3 µg A3G (blue in the top panels, 90% reduction in nERT, 99% decrease in infectivity) with WT A3F (blue in the bottom panels, 91% reduction in nERT, 99% decrease in infectivity). These findings are entirely consistent with our previous observations correlating loss of viral infection with diminished cDNA accumulation [13],[14], and indicate that APOBEC proteins are able to inhibit HIV-1 cDNA accumulation without requiring hypermutation or any target cell factors.

### Endogenous A3G Has the Same Effect on Early cDNA Accumulation as A3G Expressed in 293T Cells

**Figure 3:**
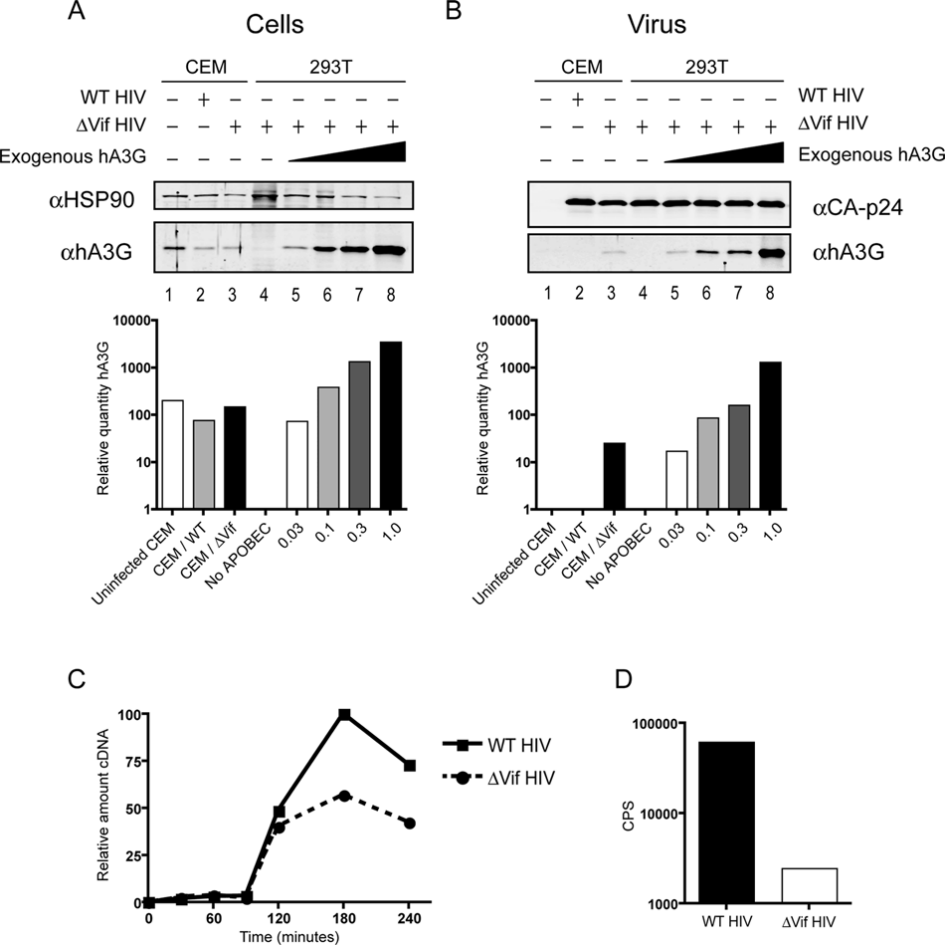
Endogenous A3G has the same effect on early cDNA accumulation as equivalent amounts of A3G expressed in 293T cells. (A) Immunoblot analysis of A3G expression in uninfected CEM cells, CEM cells infected with either WT HIV-1 or HIV-1/Δ*vif,* or 293T cells transfected with a fixed amount of HIV-1/Δ*vif* proviral plasmid (3 µg) and increasing amounts of A3G plasmid (0, 0.03, 0.1, 0.3 or 1 µg). Below the blot, the graph shows the quantity of A3G expressed relative to HSP90. (B) Viral incorporation of A3G was measured by immunoblot analysis of purified virions harvested from the cells in (A). Below the blot, the graph shows the quantity of A3G incorporated relative to HIV-1 CA-p24. (C) WT HIV-1 or HIV-1/Δ*vif* were passaged through CEM cells and used in natural endogenous reverse transcription reactions. DNA was harvested at the indicated times after the addition of dNTPs, and the relative amounts of early reverse transcription products (strong stop) were measured using quantitative PCR analysis. (D) The virus preparations from CEM cells described in (C) were used in parallel to challenge TZM β-gal indicator cells, and productive infection was measured as the induction of β-galactosidase activity, monitored using a chemiluminescent substrate. Infectivity is reported as counts per sec.

Most studies on A3G use exogenous expression systems which lend themselves to a wide variety of manipulations and can be appropriately controlled. However, it is likely that A3G is frequently expressed to higher levels in these systems than the endogenous protein is in T-cells [39]. As both the accumulation of reverse transcripts and viral infectivity decrease with increasing A3G plasmid concentration (Figure 2A and 2B), we wanted to compare the levels of A3G protein packaged into virions in these experiments with the level of endogenous A3G that is packaged. Unfortunately, we were unable to examine A3G incorporation in virions from H9 or Hut78 T-cell lines, as these cells release pelletable A3G into the medium that, in the context of infected cultures, co-purifies with viral particles (data not shown). For this reason, we passaged WT or HIV-1/Δ*vif* through another non-permissive T-cell line, CEM. While these cells do not constitutively secrete A3G, they express approximately 10-fold less A3G mRNA than H9, Hut78 or PBMCs, yet still block spreading HIV-1 replication [36]. Figure 3A shows that transfecting increasing amounts of A3G plasmid into 293T cells leads to comparative increases in A3G protein expression (lanes 4–8). This also leads to corresponding increases in the amount of A3G packaged into viral particles (Figure 3B, lanes 4–8). The amount of A3G expressed in CEM cells is equivalent to 293T cells transfected with between 0.03 and 0.1 µg A3G plasmid (Figure 3A, compare lane 1 with lanes 5 and 6). The level, as expected, is reduced in CEM cells infected with WT HIV-1 (lane 2) due to Vif expression inducing the degradation of A3G. Expression is not completely abolished as a proportion of these cells are presumably not infected at the time of sampling and therefore still express normal levels of A3G. As previously reported [5],[7],[9], WT HIV-1 particles from CEM cells do not package detectable A3G (Figure 3B, lane 2). As with the cellular expression, the amount of endogenous A3G packaged into HIV-1/Δ*vif* virions corresponds to that detected in viruses from 293T cells transfected with between 0.03 and 0.1 µg A3G plasmid (Figure 3B, compare lane 3 with lanes 5 and 6). This compares well with a previous report where HIV-1/Δ*vif* virions from PBMCs packaged the equivalent amount of A3G as viruses made in 293T cells transfected with a 1∶5 molar ratio of A3G: proviral DNA [39]. In our experiment, transfecting 0.3 µg A3G plasmid also gives approximately a 1∶5 molar ratio, and CEM cells express ∼10-fold less A3G than PBMCs, corresponding to 0.03 µg A3G plasmid.

When a nERT reaction was carried out on viruses from CEM cells, followed by qPCR for strong stop cDNA, the relative amount of viral cDNA detected in HIV-1/Δ*vif* virions also corresponded to that in viruses from 293T cells transfected with between 0.03 and 0.1 µg A3G plasmid (compare Figure 3C with Figure 2A). The reduction in cDNA levels is similar to that detected previously in CEM cells by non-PCR based methods [40]. This implies that endogenous A3G has a similar effect on cDNA accumulation as exogenously expressed A3G in 293T cells. In other words, the specific activity of the protein with respect to this attribute is similar irrespective of the cell type used for expression.

Based on the 293T titration data from Figure 2B, this modest reduction in strong stop cDNA levels in Δ*vif* viruses from CEM cells would be expected to lead to between an ∼55–90% reduction in viral infectivity. However, Δ*vif* viruses from CEM cells were slightly less infectious than this, showing ∼95% reduction in infectivity compared to WT HIV-1 (Figure 3D). This suggests that the antiviral properties of A3G may be accentuated when expressed in T-cells either through the action of cofactors provided by virus producing cells [41] or through direct effects on the protein itself [42],[43]. Regarding the former possibility, a recent report showing that endogenous A3G has significantly lower cytidine deaminase activity than A3G produced in 293T cells would indicate that any additional activities are independent of hypermutation [36].

### A3G Does Not Affect Initiation of Reverse Transcription

Several possible mechanisms could be responsible for the APOBEC-mediated decrease in the accumulation of HIV-1 strong stop cDNA. Whilst rapid degradation is still a formal possibility, we have shown that it would have to occur in the absence of hypermutation and be driven entirely by components packaged into the virion from the producer cell. The alternative explanation posits that A3G inhibits the synthesis of cDNA. This could be achieved by A3G inhibiting the annealing of the tRNA^lys3^ primer to the primer binding site, preventing reverse transcriptase from recognising the primer and initiating reverse transcription, inducing a specific block (i.e., at a particular site) after initiation of reverse transcription but before the completion of strong stop, or by imparting a general decrease in the processivity of reverse transcriptase itself. To begin to distinguish between these possibilities, we decided to look at the initiation of reverse transcription by setting up a novel assay system.

**Figure 4:**
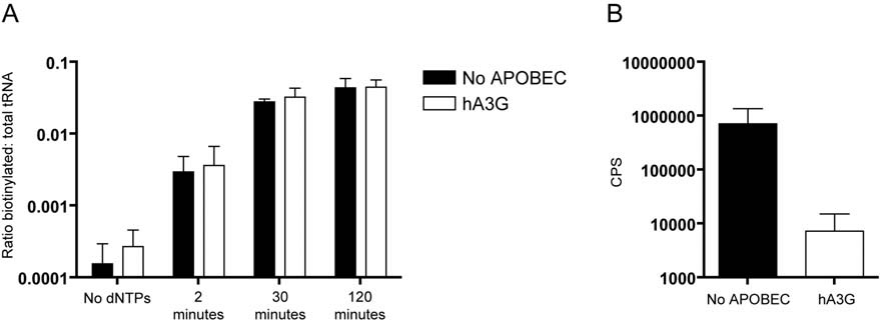
A3G does not affect initiation of reverse transcription. (A) Equivalent amounts of HIV-1/Δ*vif* produced from 293T cells in the presence or absence of human A3G were incubated with biotinylated-dCTP during modified natural endogenous reverse transcription reactions. Samples were taken at 2, 30, or 120 min after the addition of biotinylated-dCTP, and at 120 min for the control reaction with no dNTPs. Reverse transcription initiation was assessed by measuring both the amount of biotinylated primer tRNA^lys3^ that bound to a streptavidin-coated 384-well qPCR plate and the total amount of tRNA^lys3^ purified from each reaction. The ratio of biotinylated tRNA^lys3^ to total tRNA^lys3^ is plotted on the y-axis. Error bars show the standard deviation of four independent viral preparations. (B) The same virus preparations described in (A) were used in parallel to challenge TZM β-gal indicator cells, and productive infection was measured as the induction of β-galactosidase activity, monitored using a chemiluminescent substrate. Infectivity is reported as counts per sec.

As before, HIV-1/Δ*vif* viruses were made in 293T cells in the presence or absence of A3G and nERT reactions were performed. In these reactions, however, the mix of dNTPs was replaced with just biotinylated dCTP: cytidine being the first base added to the tRNA^lys3^ primer. If the tRNA^lys3^ primer was correctly bound to the genomic RNA, and reverse transcription could be initiated, the addition of dCTP would result in the tRNA^lys3^ primer becoming biotinylated. Total RNA from each time point was purified and an aliquot was incubated in streptavidin-coated qPCR plates. This allowed biotinylated dCMP-tRNA to bind specifically to the plate. A set of qPCR standards and an aliquot of total purified RNA for each sample was then added to the plate as controls, and a one-step quantitative reverse-transcriptase PCR was performed for tRNA^lys3^. The results are shown in Figure 4A. The amount of total tRNA^lys3^ was constant for all samples (data not shown), indicating each sample had received equal input virus and all RNA purification steps were similarly efficient. The amount of tRNA^lys3^ in the bound samples however, increased with time from trace amounts in the control samples to substantial levels at 30 min, continuing to increase slightly to 2 h (data not shown). The ratio of biotinylated (bound) tRNA^lys3^ to total tRNA^lys3^ therefore also increased with time (Figure 4A), implying biotin was being efficiently added to the tRNA^lys3^ primer during the nERT reaction. Importantly, although the presence of A3G significantly decreased viral infectivity (Figure 4B), we were unable to detect any difference in the amount of tRNA biotinylation between viruses made in the presence or absence of A3G. This suggests that A3G does not inhibit either the placement of the tRNA^lys3^ primer or the initiation of reverse transcription from that primer.

### The Decrease in cDNA Levels Is Greater with Increasing Elongation

**Figure 5:**
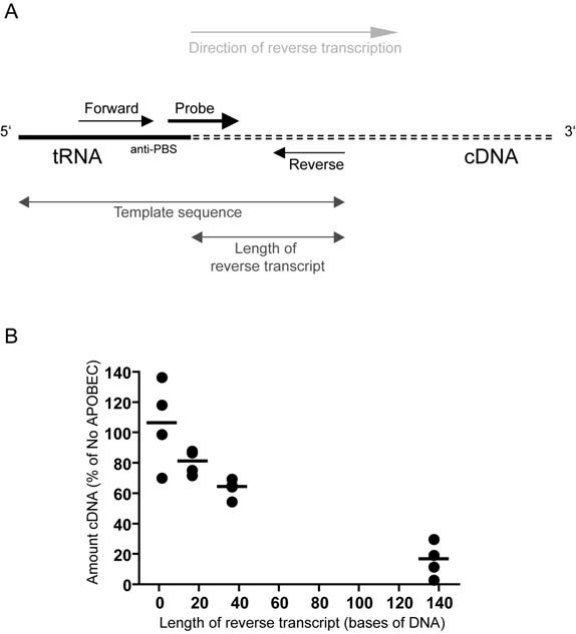
A3G inhibits the accumulation of longer reverse transcription products more than shorter ones. (A) Schematic to show how the length of the reverse transcript was measured for each primer/probe set. The exact positions of the primers and probe are not depicted. (B) Natural endogenous reverse transcription reactions were performed for 2 h with HIV-1/Δ*vif* produced in the presence or absence of A3G. Quantitative PCR was carried out with different primer/probe sets to measure the amount of viral cDNA of the various lengths indicated. The amount of cDNA in virions with A3G is plotted as a percentage of that in virions without A3G. Data from Figure 4 are included to show the relative amount of reverse transcripts of 1 base in length. Each point represents an independent pair of viral preparations. Lines indicate the mean value.

As the initiation of reverse transcription is normal in the presence of A3G, it is possible that A3G inhibits the ensuing elongation of reverse transcripts, either continuously or at a specific point(s) during early cDNA synthesis. Initially, we attempted to visualise early reverse transcription products on polyacrylamide gels after performing nERT reactions using ^32^P-labelled dNTPs. However, this was not sensitive enough to detect small cDNA fragments, other than strong stop DNA, even in the absence of A3G (data not shown). Therefore, to investigate the effect on cDNA elongation, we designed qPCR primers and probes for monitoring different regions within the strong stop cDNA fragment. For the purpose of this analysis, we assumed that if a product amplified, then the template sequence must reach the 5′ end of the reverse primer for each primer/probe set (Figure 5A). The length of the reverse transcript was therefore taken as the number of bases from the 3′ end of the anti-primer binding site (anti-PBS) to the 5′ end of the reverse qPCR primer (Figure 5A). Our standard strong stop primers amplify a region from R into U5, implying any reverse transcripts detected with this primer/probe set are at least 137 bases long. Our new primer/probe sets amplified reverse transcripts of 16 or 36 bases. As these primer/probe sets amplified templates that were part RNA, part DNA, a reverse transcription step was carried out before qPCR to ensure efficient amplification by DNA polymerase.

Viruses were synthesized with or without A3G as before and nERT reactions were performed. Samples were taken after 2 h, added to cells-to-signal lysis buffer and an aliquot of the lysate was taken directly to a one-step quantitative reverse-transcriptase PCR. The amount of each cDNA detected in virions in the presence of A3G was calculated as a percentage of that detected in the absence of A3G. This was plotted against the length of the reverse transcript amplified by each primer/probe set (Figure 5B). Data from independent preparations of pairs of viruses are plotted as individual points to illustrate the experimental variation and the mean for each primer/probe set is shown by a line. It is clear that the amount of cDNA detected in the presence of A3G decreased with increasing distance from the tRNA^lys3^ primer. There was only an ∼20% decrease in the amount of cDNA reaching 16 bases, increasing to an ∼35% decrease in the amount of cDNA reaching 36 bases, and an ∼85% decrease in cDNA 137 bases long. Thus, A3G appears to inhibit the elongation of reverse transcripts.

Importantly, these effects on cDNA synthesis under nERT conditions are specific for the viral RNA template rather than directly inhibiting reverse transcriptase itself as reactions performed using viral lysates, exogenous poly-rA template and oligo-dT primer were unaffected by the presence of A3G in virions (data not shown).

## Discussion

Members of the APOBEC family of cytidine deaminases inhibit HIV-1 infection. However, there are still many uncertainties and controversies as to exactly how they exert their anti-viral effects. Before the identification of A3G, it was known that *vif*-deficient HIV-1 produced less provirus in non-permissive cells [44]–[47], and this has been confirmed in recent studies using A3G expression vectors [11], [13]–[20],[31]. There are two ways this defect could occur; either reverse transcripts could be synthesized but then degraded before they can form integrated provirus, or there could be a block to DNA synthesis. The former mechanism was initially favoured by many since the APOBEC mediated appearance of uracils in DNA could serve as the signal for recognition by cellular DNA repair enzymes and nucleases. However, anti-viral activity is not always associated with hypermutation, (reviewed in [3]), and the enzymes responsible for recognising and removing uracils in DNA are apparently expendable for anti-viral function [16],[17],[21]. Moreover, deaminase defective variants of APOBEC proteins are still able to reduce the accumulation of viral cDNA [14], suggesting that antiviral effects can be achieved by a non-editing mechanism(s). While this effect was first noted with A3G, we have shown that it is more pronounced for A3F, as non-editing A3F mutants display antiviral phenotypes that closely match the wild-type protein over a range of concentrations [14]. Accordingly, since we have previously shown that the accumulation of reverse transcripts correlates well with viral infectivity, we set out to investigate this effect further.

Initially, we measured cDNA accumulation in different target cells (Figure 1). Similar results were seen in both permissive and non-permissive cell types, indicating that the presence of endogenous A3G in the target cell causes no additional inhibition, even in activated PBMCs. Moreover, this implied either that any target cell factors involved in diminishing cDNA accumulation are present in all cell types studied or that these effects do not require any target cell factors. To examine this, we established a natural endogenous reverse transcription (nERT) assay and found that the extent of inhibition was highly reminiscent of that seen in target cell infections (Figure 2A), revealing that no target cell factors are necessary for the block to cDNA accumulation. Whilst this does not refute a degradative mechanism for cDNA turnover, it does eliminate proteasomal degradation and implies that any endo- or exonucleases involved must be copurified or packaged into viral particles. As cDNA levels were also reduced by deaminase deficient APOBEC variants (Figure 2C), an alternative trigger for degradation other than uracils in DNA would be necessary. Theoretically, A3G could cause the specific packaging of an as yet unidentified nuclease responsible for degrading nascent reverse transcripts. However, there is no direct evidence in the literature for A3G-induced degradation and, as we expand upon below, this is not our preferred explanation.

The fact that the block to cDNA accumulation can be detected in nERT reactions allowed us to investigate this process in ways that are not possible in cells, but resemble HIV-1 infections more closely than reconstitution studies using purified components. For instance, we were able to monitor the initiation of reverse transcription in virions (Figure 4). Recently, the Kleiman group have reported that both A3G and A3F prevent tRNA^lys3^ primer annealing *in vitro* via an interaction with nucleocapsid [19],[31],[48]. However, annealing was not measured directly, and in a very thorough recent study, Iwatani et al., found no effect on primer placement or initiation of the +1 cDNA, again *in vitro* using purified components [15]. By using a novel variation of the nERT assay to study reverse transcription initiation in viral particles, we have shown that A3G does not affect the addition of a biotinylated dCTP first base to the tRNA^lys3^ primer. We acknowledge that some reverse transcription may have initiated prior to incubation with biotinylated dCTP, but as we detect more than a two log increase in biotinylation after 30 min, we assert that this represents significant initiation in our experiment.

Although there was no block to the initiation of reverse transcription, we could detect a significant reduction in the level of strong stop cDNA in cells (Figure 1) and nERT reactions (Figures 2 and 5) in the presence of A3G. This indicates either that there is an early block to reverse transcription or that cDNA is degraded very rapidly. To address whether reverse transcription was inhibited at a particular position or whether elongation was progressively impeded, we designed new qPCR primer/probe sets to measure reverse transcripts shorter than the 137 nucleotide standard strong stop product. The amounts of cDNA detected in the presence of A3G decreased with increasing distance from the tRNA^lys3^ primer (Figure 5B), from an ∼20% reduction in cDNA reaching 16 bases to an ∼35% decrease at 36 bases and an ∼85% decrease at 137 bases. Some variation was seen between independent virus preparations (indicated by individual points) most likely reflecting minor differences in A3G expression and packaging. From these data, it therefore appears that there is no single abrupt point of termination to reverse transcription.

The results of our study are in complete agreement with a recent report from Iwatani et al. who show that purified recombinant A3G is able to inhibit the elongation of exogenous cDNA by HIV-1 reverse transcriptase *in vitro* [15]. They hypothesise that A3G binding to HIV-1 RNA or single stranded DNA physically blocks RT movement along the template. If A3G bound genomic RNA at random points, this would imply that the likelihood of synthesizing a given product would decrease with increasing product length, as borne out here in Figure 5. The fact that A3G has been shown to bind several different RNA molecules implies that binding is not particularly sequence specific [6], [28], [49]–[52]. Work on HBV has recently revealed that A3G is also able to inhibit the early steps in minus-strand DNA synthesis in this virus via a block to DNA strand elongation [33].

Other groups have reported a less dramatic decrease in early products than we see here, with escalating reductions at progressively later stages of replication [17],[18],[20]. Indeed, we have also published a greater decrease in the levels of later products compared to early transcripts [14]. The difference in the magnitude of the early effect between different groups has been attributed to differences in A3G expression levels, and we concur with this view since we show clearly that both the levels of early cDNA accumulation and infectivity titrate down with ascending A3G concentration (Figure 2A and 2B).

Whilst the levels of endogenous A3G expression and viral incorporation were low in CEM cells, the effect on early cDNA accumulation was consistent with that seen with exogenous A3G (Figures 2 and 3). We note, however, that the relative deficit in infectivity imparted by APOBEC proteins in CEM cells is greater than anticipated on the bases of the nERT assay. Unfortunately, we found the efficiency of strand transfer and second strand synthesis to be very low in nERT reactions, and therefore it was not possible to use this system to investigate the effect on later steps in reverse transcription with any degree of quantification or certainty (data not shown). Possible underlying reasons are discussed above, and are a subject for future investigations. Importantly, a mode of action that involves direct binding of A3G to viral RNA would be expected to be sensitive to A3G concentration: if A3G can bind to multiple regions of the RNA genome with equal preference, then the probability of binding to a region within R or U5, and therefore blocking strong stop cDNA synthesis, would increase with A3G concentration. Theoretically, a single molecule of A3G may be all that would be required to inhibit the synthesis of a full length reverse transcript, such that even low levels of endogenous A3G would be sufficient to prevent viral infection.

Based on recent findings, we suggest that the relative contribution of editing and non-editing effects to the antiviral phenotype of A3G may depend upon its circumstances. If the inhibition of cDNA synthesis is inefficient, the resulting nascent transcripts may still be inactivated via hypermutation and loss of genetic integrity, and the production of such mutated viral sequences has been well documented in both cultured cells and *in vivo* [7], [10]–[12], [35], [53]–[56]. Interestingly, the editing capability of endogenous A3G in cultured T-cells appears to be reduced compared to A3G produced in 293T cells [36]. In addition, viral cDNAs recovered from PBMCs infected with HIV-1/Δ*vif* harbour lower levels of G-to-A mutations compared to cDNAs from H9 cells infected with HIV-1/Δ*vif*, both in terms of the number of clones carrying mutations and the number of mutations per clone [57]. This might suggest a lesser role for hypermutation *in vivo*, although this issue requires further investigation. Nevertheless, the capacity to suppress HIV-1 infection via dual mechanisms presumably serves to enhance the potency of this class of antiviral proteins.

Recently, two groups have shown that HIV-1/Δ*vif* can replicate in stable cell lines expressing deaminase-defective A3G mutants but not wild-type A3G [58],[59]. They have interpreted these results as proving that deamination is essential for A3G activity. We suggest that such observations should be interpreted with caution as we have shown that the A3G E259Q mutant also has a substantially reduced ability to inhibit cDNA accumulation (Figure 2 and [14]). An alternative explanation for these findings is that disruption of the deaminase motif of A3G also affects an attribute necessary for inhibiting reverse transcription; since the C-terminal deaminase domain of A3G must engage nucleic acids for the purpose of editing, one possibility is that this defect could be in RNA binding. At present, it is unclear what effects such mutations have on the protein structure and function.

In this study, we have shown that A3G-mediated inhibition of cDNA accumulation occurs independent of both the target cell and hypermutation. Although there is no defect in tRNA^lys3^ priming of reverse transcription, a small decrease in the amount of cDNA can already be observed at 16 bases, and this reduction increases with distance from the tRNA^lys3^ primer. These data bolster the argument against a degradative mechanism being responsible for the decreases in viral cDNA that are observed in the presence of A3G, and support the proposal that A3G inhibits the elongation of nascent HIV-1 cDNA by steric hindrance of reverse transcriptase through direct binding to viral genomic RNA.

## Materials and Methods

### Plasmid Constructs

All APOBEC proteins were expressed from pcDNA3.1 as previously described; for WT human A3G [1], A3G E259Q [27], WT A3F and A3F E251Q [14]. Expression vectors for wild type and Δ*vif* HIV-1 (pIIIB and pIIIB/Δ*vif*, respectively), have been described before [35], and contain a mutation at nucleotide 567 of the provirus to create a G to A substitution in the U5 region of the 5′-LTR, that would copy to the 3′-LTR during reverse transcription.

### Cells and Virus Production

All cell lines were maintained under standard conditions. Δ*vif* HIV-1 stocks were typically prepared by co-transfection of 293T cells with proviral plasmid and A3G expression plasmid (or empty pcDNA3.1 vector) at a ratio of 3∶1. For the A3G titration experiment shown in Figure 2, 3 µg of Δ*vif* provirus was co-transfected with varying amounts of A3G plasmid as indicated. For the experiment that included A3F and A3G/A3F mutants (Figure 2) a ratio of 1∶1 provirus to APOBEC expression vector was used. The media was changed ∼18 h after transfection and replaced with DMEM containing 20 µl/ml RQ1-DNase (Promega). After 8 h, the cells were washed and fresh media was added for a further 17 h, before virus containing supernatants were harvested. To study endogenous A3G, wild-type or Δ*vif* HIV-1 pseudotyped with the G-protein from vesicular stomatitis virus was made in 293T cells and used to infect CEM cells by spin infection at 1200×g for 2.5 h at 20°C. The cells were extensively washed and allowed to grow for 24 h before the media was changed again, and virus containing supernatants were harvested 16 h following this. All viral titres were quantitated by enzyme-linked immunosorbent assay (ELISA) for p24^CA^ content.

### Viral Infections

Viral infectivities were determined in single-cycle assays by challenging 10^5^ TZM-β-gal indicator cells [60] with viruses corresponding to 5 ng p24^CA^, and measuring the induced expression of β-galactosidase activity in cell lysates after ∼28 h, using the Galacto-Star system from Applied Biosystems.

For quantitative PCR analysis of cDNA in target cells, viral preparations containing 30 ng of p24^CA^ were added to 10^6^ SupT1, CEM-SS or CEM cells or PBMCs rotating at 4°C for 2 h. Cells were then washed, resuspended in fresh RPMI media, and incubated at 37°C for 0–24 h. Total DNA was purified using the DNeasy kit from Qiagen, and eluted in a total volume of 200 µl. After treatment with *Dpn1* for 2 h at 37°C, 2 µl of DNA was analysed by real-time quantitative PCR.

### Natural Endogenous Reverse Transcription Assays (nERT)

Viral preparations containing 10–25 ng of p24^CA^ were pelleted by centrifugation for 1 h at 20,000×g at 4°C. Virions were resuspended in PBS, 2.5 mM MgCl_2_, 15 µg/ml melittin and 1 mM dNTPs, and incubated at 37°C. Control reactions were incubated without any dNTPs for the same length of time as the longest time point. At the specified time points, aliquots were removed and equal volumes of PBS containing 40 µg/ml “carrier” salmon sperm DNA added. DNA was then purified using using the DNeasy kit from Qiagen, and eluted in a total volume of 200 µl. After treatment with *Dpn1* for 2 h at 37°C, 2 µl of DNA was analysed by real-time quantitative PCR. For later experiments, an equal volume of 2× Cells-to-signal lysis buffer (Ambion) was added instead of PBS with carrier DNA, and samples were diluted 1∶10 in water before analysing 2 µl directly in real-time quantitative PCR.

### Reverse Transcription Initiation nERT Assay

For this assay, a nERT reaction was performed essentially as above, with the exception of the dNTP addition: pelletted virions were resuspended in PBS, 2.5 mM MgCl_2_, 15 µg/ml melittin and 0.5mM biotin-11-dCTP (Jena Bioscience), and incubated at 37°C. Control reactions were incubated without any dNTPs for 2 h. At the specified time points, aliquots were removed, added to QIAzol lysis buffer (Qiagen) and frozen at −80°C. RNA was purified using the Qiagen miRNeasy kit and eluted in 30 µl of water. 2 µl 10× PBS was added to 18 µl RNA to make the final samples 1×PBS and the samples were heated at 95–100°C for 3 min and cooled instantly on ice to separate tRNA from genomic RNA. Samples were then added to streptavidin coated, 384-well, Strep Thermo-Fast plates (Abgene) and incubated at 37°C for 30 min. Plates were extensively washed and all liquid aspirated. Standards and aliquots of total purified RNA were added to the plates before the addition of Ag-path-ID One-Step RT-PCR Kit reagents (Ambion) and tRNA^lys3^ levels were measured by quantitative PCR analysis.

### Quantitative PCR

Strong stop reverse transcription products were detected using primers that amplify the region between nucleotides 500 and 635 of the provirus: oHC64 (5′-TAACTAGGGAACCCACTGC) and oHC65 (5′-GCTAGAGATTTTCCACACTG) with probe oHC66 (5′-FAM-ACACAACAGACGGGCACACACTA-TAMRA). Reactions were performed in triplicate, in TaqMan Universal PCR master mix (UNG-less) using 900 nM of each primer, and 250 nM probe. After 10 min at 95°C, reactions were cycled through 15 sec at 95°C followed by 1 min at 60°C for 40 repeats, carried out on an ABI Prism model 7900HT (Applied Biosystems). The Δ*vif*-HIV-1 expression vector, pIIIB/Δ*vif*, was diluted into purified SupT1 cellular DNA to create a series of control samples that were used to calculate relative cDNA copy numbers and confirm the linearity of the assay.

tRNA^lys3^ was detected using primers tRNA-15for (5′-GTCGGTAGAGCATCAGACTTTTAATCT) and Long PBS-G-(C)7rev (5′-CCCCCCCGTGGCGCCCGAACAGGGACTTGAAC) with MGB probe Probe-tRNA43for (5′-FAM-AGGGTCCAGGGTTC-MGB). An oligo with sequence: 5′-CCCCCCCGTGGCGCCCGAACAGGGACTTGAACCCTGGACCCTCAGATTAAAAGTCTGATGCTCTACCGAC was serially diluted in water to create a standard curve. Note that this primer/probe set was originally designed to detect tRNA^lys3^ with a 3′ extension. One-step quantitative reverse transcription PCR was performed using the Ag-path-ID One-Step RT-PCR Kit from Ambion using 20 µl reaction volumes and final primer and probe concentrations of 450 nM and 125 nM respectively. Cycling conditions were 45°C for 15 min then 95°C for 10 min, followed by 40 cycles of 15 sec at 95°C and 45 sec at 60°C.

Earlier products of reverse transcription either 16 or 36 bases from the 3′ end of the tRNA^lys3^ primer were detected with set 2 or set 3 primer/probe sets respectively. Set 2 primer sequences were: tRNA-15for and PBSU5-rev, (5′-GGAAAATCTCTAGCAGTGGCG) with MGB probe Probe-tRNA43for. Set 3 primer sequences were tRNA-41for (5′-TGAGGGTCCAGGGTTCAAGT) and U5-rev-3 (5′-CAGACCCTTTTAGTCAGTGTGGAA) with probe Probe-PBSU5f-3 (5′-FAM-CCTGTTCGGGCGCCACTGCT-BHQ1). An oligo with sequence: 5′GTCGGTAGAGCATCAGACTTTTAATCTGAGGGTCCAGGGTTCAAGTCCCTGTTCGGGCGCCACTGCTAGAGATTTTCCACACTGACTAAAAGGGTCTGAGGGATCTCTA was serially diluted in water to create a standard curve. One-step quantitative reverse transcription PCR was performed using the Ag-path-ID One-Step RT-PCR Kit using 20 µl reaction volumes and final primer and probe concentrations of 400 nM and 120 nM respectively. Cycling conditions were as above for tRNA^lys3^.

All primer and probe sequences, as well as their respective amplicons, are also provided in Table S1.

### Immunoblot Analysis

Virions were concentrated by centrifugation through a 20% (w/v) sucrose cushion before immunoblot analysis. Human A3G protein, heat shock protein (HSP) 90 or viral p24^CA^ proteins were detected in whole cell lysates of uninfected or HIV-1 infected CEM cells or transfected 293T cells, or in virion lysates, using a polyclonal rabbit serum to A3G, HSP 90α ~β (H-114, Santa Cruz Biotechnology), or 24-2, a monoclonal anti-CA antibody, respectively, secondary antibodies IRDye800CW Goat Anti-rabbit and IRDye800CW Anti-mouse (LI-COR Biosciences UK Ltd) and Li-cor Odyssey infrared imaging and quantitation.

## Supporting Information

Table S1 Names and sequences of qPCR primers and probes used.
(0.04 MB PDF)
