# Supplementary material for: APOBEC3G Inhibits Elongation of HIV-1 Reverse Transcripts
Source: PLoS Pathog. 2008 Dec 5;4(12):e1000231. doi: 10.1371/journal.ppat.1000231 (PMC2584787; doi:10.1371/journal.ppat.1000231)
Supplement: Table S1 — Names and sequences of qPCR primers and probes used. (0.04 MB PDF) [file ppat.1000231.s001.pdf]

**Strong stop** (137 bases)[a]		
Forward Primer	oHC64	5' TAACTAGGGAACCCACTGC
Reverse Primer	oHC65	5' GCTAGAGATTTTCCACACTG
Probe	oHC66	5' FAM-ACACAACAGACGGGCACACACTA-TAMRA
**tRNA^{lys3}** (1 base)[a]		
Forward Primer	tRNA-15for	5' GTCGGTAGAGCATCAGACTTTTAATCT
Reverse Primer	Long PBS-G-(C)7rev	5' CCCCCCCGTGGCGCCCGAACAGGGACTTGAAC
Probe	Probe-tRNA43for	5' FAM-AGGGTCCAGGGTTC-MGB
**Set 2** (16 bases)[a]		
Forward Primer	tRNA-15for	5' GTCGGTAGAGCATCAGACTTTTAATCT
Reverse Primer	PBSU5-rev	5' GGAAAATCTCTAGCAGTGGCG
Probe	Probe-tRNA43for	5' FAM-AGGGTCCAGGGTTC-MGB
**Set 3** (36 bases)[a]		
Forward Primer	tRNA-41for	5' TGAGGGTCCAGGGTTCAAGT
Reverse Primer	U5-rev-3	5' CAGACCCTTTTAGTCAGTGTGGAA
Probe	Probe-PBSU5f-3	5' FAM-CCTGTTCGGGCGCCACTGCT-BHQ1

Table S1. Names and sequences of qPCR primers and probes used.
[a] Measured number of bases of cDNA from the 3' end of the tRNA^{lys3} primer.
